# Status and advancement of root-knot nematode management strategies and the emerging CRISPR/Cas biotechnology application

**DOI:** 10.1007/s10142-025-01804-w

**Published:** 2026-02-07

**Authors:** Xiaoping Pan, Ugur Yildiz, Sarah K. Armstrong, Kaitlyn Bissonnette

**Affiliations:** 1 Department of Biology, East Carolina University, Greenville, NC 27858 USA; 2 Cotton Incorporated, Cary, NC 27513 USA

**Keywords:** Root-knot nematodes, Nematode resistance, Genome editing, CRISPR/Cas9, Crop improvement, Cotton

## Abstract

Root-knot nematodes (RKNs), *Meloidogyne spp*., exhibit a broad host range, threatening more than 3000 species of plants, including agriculturally important crops such as cotton (*Gossypium hirsutum*), tomato (*Lycopersicon esculentum*) and rice (*Oryza sativa*). Among the over 90 RKN species, the four most prevalent are *M. incognita*, *M. arenaria*,* M. javanica*, and *M. hapla*, with *M. incognita* being the most damaging. This paper reviewed the current RKN management strategies, including chemical nematicides, biological control, crop rotation, and resistant varieties, with a focus on the application of the revolutionary CRISPR/Cas genome editing tool in developing RKN resistance in plants. CRISPR/Cas has been widely utilized for improving crop traits due to its specificity, streamline, and inheritability. Recent progress has demonstrated the simplicity and robustness of CRISPR/Cas technology in improving plant traits. Among these, the development of nematode resistance by CRISPR/Cas knocking out of plant compatibility factors in model and commercial plants, has achieved significant progress. This review summarizes the RKN parasitism mechanisms and plant compatibility factors that would be promising CRISPR/Cas targets. The fundamentals and key aspects of CRISPR/Cas genome editing technology are addressed and discussed, and an example experimental pipeline for developing nematode resistance in cotton is described.

## Introduction

**Fig. 1:**
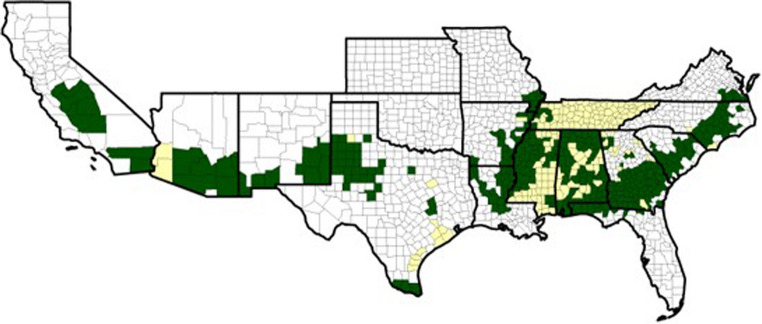
Root-Knot nematode population distribution and density by National Cotton Council (National Cotton Council 2009)

Plant-parasitic nematodes (PPNs) are major plant pests, causing massive economic loss estimated at around $100–157 billion annually and accounting for a significant 8.8–14.6% of crop losses in the tropical and subtropical climate region (Abad et al. 2008; Azlay et al. 2023). According to multi-year field surveys mostly conducted by USDA-ARS (Agriculture Research Service) scientists and extension specialists who documented yield losses in paired infested/non-infested fields, the root-knot nematode (RKN) and reniform nematode are the most damaging nematodes in the US cotton belt, from California to North Carolina (Fig. 1). Rising greenhouse gases are expected to increase global temperature and change water availability, which in turn will affect the conductivity of environmentally sensitive pathogens and the susceptibility of plants to diseases, including nematode infections (Velásquez et al. 2018). As a result, hosts and parasites are experiencing shifts in their thermal environment, and host-parasite population dynamics will be affected, especially if host performance is negatively impacted by heating (Claar and Wood 2020; Hector et al. 2023). RKN development and reproduction rates are temperature-dependent; warmer soils accelerate egg hatching, juvenile development, and the time to reproduction. Shorter life cycle leads to higher population density within a single season (Dutta and Phani 2023).

## Root-knot nematode biology

Root-knot nematodes (*Meloidogyne spp.*, mainly *M. incognita*, *M. javanica*, *M. arenaria*, and *M. hapla*) are among the most destructive PPNs infecting over 3000 plant species, including important crops such as cotton (*Gossypium hirsutum*), tomato (*Lycopersicon esculentum*), and rice (*Oryza sativa*) (Eisenback and Triantaphyllou 1991; Ounis et al. 2024). The infection starts when the second-stage juvenile (J2) enters plant roots via chemotaxis. The J2 penetrates the root, migrates, and establishes a permanent feeding site by injecting cell wall-degrading enzymes and other effectors to induce nuclear division without cytokinesis. Specifically, *M. incognita* J2s enter the root at the elongation zone, migrate to the tip, and then turn to enter the vascular cylinder and proceed upwards until they reach the differentiation zone, where they generate numerous hypertrophied giant cells (GCs) from plant parenchyma cells (Eisenback and Triantaphyllou 1991). As J2s continuously obtain nutrients through GCs, they become sedentary inside the root and molt through J3, J4, and eventually develop into sexually dimorphic adult females or males. Females become sessile and lay eggs on the root surface, while males may leave the root. *M. incognita* reproduces via mitotic parthenogenesis, meaning females produce offspring without fertilization by sperm. Under favorable conditions, a single female can produce around 500 eggs. These eggs are deposited in a desiccation-resistant gelatinous matrix. The life cycle of *M. incognita* is temperature-dependent and lasts approximately 3–4 weeks under optimal conditions. Warm, sandy soils favor the survival and development of *M. incognita* egg masses. (Eisenback and Triantaphyllou 1991; Moens et al. 2009; Dávila-Negrón and Dickson 2013).

## Host-nematode interactions

Root-knot nematodes are highly adapted parasites which effectively manipulate plant physiology/compatible factors for its development and reproduction. Plant compatibility factors are functional machinery that nematodes require for successful parasitism, which include plant susceptible genes (S genes), transcription factors, protein/enzymes and modifications, cellular machinery, and coordinated tissue structure. RKNs secrete a cocktail of effector proteins to manipulate hosts and establish feeding sites (Williamson and Gleason 2003, Khan and Khan 2021). Mainly produced from the subventral and dorsal esophageal gland cells, effectors and small molecules are injected into host tissues via a syringe-like stylet. Zhou et al. found that Minc03329 transcript expressed in the subventral esophageal glands of *M. incognita* pre-parasitic J2s and was significantly up-regulated in parasitic J2s (Zhou et al. 2023a, b). RKN excreted effectors play crucial roles in modifying plant cell walls, establishing and maintaining feeding sites, suppressing host defensive immune response, and facilitating continuous nutrient uptake for nematode development (Mejias et al. 2019; Vieira and Gleason 2019). First, plant cell wall components are targeted by PPNs; For example, *M. incognita* secretes pectolytic enzymes such as xylanases to degrade pectin and other plant cell wall components (Mitreva-Dautova et al. 2006; Haegeman et al. 2012). Cell wall modifiers include β−1,4-endoglucanases, pectate lyases, and expansins. They dissolve and loosen cell walls by degrading cellulose and pectin (Glass et al. 2015).

To establish and maintain their feeding sites, RKNs manipulate basic plant metabolic components for their own advantage. Oppermann et al. observed that a root-specific gene, *TobRB7*, which encodes a water channel protein (aquaporin), is specifically expressed within the developing giant cells induced by RKNs infection. This pioneering study demonstrated that RKNs actively direct the expression of this plant gene. The high expression of *TobRB7* may serve the nematode’s needs by increasing the flow of water and solutes into giant cells, thereby supporting nematodes’ rapid expansion and the high metabolic activity (Oppermann et al. 1994). Xue et al. also demonstrated that the effector Mi8D05, expressed in subventral esophageal gland cells of *M. incognita* J2s, was delivered to the apoplast of root cells during infection. They interact with plant aquaporin tonoplast intrinsic protein 2 (TIP2) and may play an important role in helping water and solute transport to giant cells. RNAi knockdown of Mi8D05 significantly reduced *M. incognita* infection by up to 90% compared to the control (Xue et al. 2013). To maintain the feeding site, sugar transporters like SWEET transporters are susceptible to hijacking by RKN, and their expression levels are induced upon *M. incognita* infection (Zhou et al. 2023a, b). These transporters facilitate the efflux of carbohydrates from plant cells, a process critical for functions like phloem loading and nectar secretion, which make them prime targets for nematodes seeking a nutrient source (Hu et al. 2021).

Plants innate immunity employed two layers of immunity against pathogens and other stressors: pattern-triggered immunity (PTI) and effector-triggered immunity (ETI) (Jones et al. 2006, Cui et al. 2015). PTI is the first layer of response, conducted by plant cell pattern-recognition receptors (PRRs). Upon recognition of pathogens, PRRs activate various defensive responses, including activation of various signaling cascades and defense response genes, reactive oxygen species (ROS) burst, callose barrier formation, etc. PTI is a basal defense response against most conserved pathogens, while ETI is the second-line immune response, often more specific, robust, and faster (Abad and Williamson 2010; Lin et al. 2016; Yuan et al. 2021). Plants that are resistant to RKNs contain R genes that encode NLR (nucleotide-binding domain and leucine-rich repeat) receptors. These receptors specifically recognize the nematode’s effectors and activate ETI. A classic example is the Mi gene in tomato (Milligan et al. 1998; Branch et al. 2004; Williamson and Kumar 2006; Kaloshian and Teixeira 2019,). It detects nematode effectors and activates hyper-sensitive responses like programmed cell death and phloem-based necrosis, to create a toxic, nutrient-poor environment to starve nematodes and prevent the establishment of a feeding site. Nematode effectors act by interfering with PTI and ETI. Lin et al. reported that the transthyretin-like protein MjTTL5 is an important effector secreted by *M. javanica*, which interacts with *Arabidopsis* ferredoxin to enhance the plant reactive oxygen species (ROS)-scavenging activity, thus suppressing the ROS-triggered plant defense response (Lin B et al. 2016). Similarly, the C-type lectine (CTL) Mg01965 effector secreted by *M. graminicola* into the plant apoplast facilitates nematode parasitism by reducing plant ROS production at the early stage of nematode infection. Knockdown of Mg01965 in infective juveniles by RNAi in vitro significantly reduced its parasitism success (Zhuo et al. 2019). In contrast, the C-type lectin (CTL)-like effector MiCTL1 secreted by *M. incognita* reduced *Arabidopsis* catalases (CATs) activity and downregulated stress-response gene expression, making it more susceptible to infection (Zhao et al. 2021).

Hormone-mediated signaling pathways also regulate plant defense responses to abiotic and biotic stress including *Meloidogyne* infection (Sikder et al. 2021; Prakash et al. 2023; Umer et al. 2023). Exogenous supply of jasmonate (JA) and ethephon (ET) induced a strong defensive response in roots companied by upregulation of defense-response genes (Fujimoto et al. 2011; Nahar et al. 2011; Umer et al. 2023). The *WRKY* transcription factors are regulated by phytohormones, including SA, cytokinin, and auxin (Berry and Argueso 2022). Chinnapandi et al. found that expressions of *WRKY45* increased at the early stage of *M. javanica* infection and were highly expressed in feeding cells until gall maturation. Thereby, the *WRKY* genes are considered one of plant compatibility factors to *Meloidogyne* parasitism (Chinnapandi et al. 2017). Table 1 lists plant compatibility factors interacting with nematode effectors that would potentially serve as targets for gene silencing using advanced biotechnology, including the CRISPR/Cas genome editing (Table 1). Knocking out or silencing of plant compatibility factors can lead to increased plant resistance.

**Table 1:** Nematode effectors and corresponding plant compatibility factors

Nematode effectors	Meloidogyne spp	Plant species	Plant compatibility factors/targets and outcomes	References
Mi16D10	*M. incognita*	Arabidopsis	Plant transcription factor; stimulate root growth	Huang et al. 2006
Mi8D05	*M. incognita*	Arabidopsis	aquaporin tonoplast intrinsic protein 2 (TIP2); giant cell maintenance	Xue et al. 2013
Mi-CRT	*M. incognita*	Tomato	immune suppressor ROT1; Suppress Program cell death (PCD), facilitate gall formation	Jaouannet et al. 2013
Mi-7H08	*M. incognita*	Arabidopsis	Activate transcription in nuclei	Zhang et al. 2015
MiMSP40	*M. incognita*	Arabidopsis	Suppress PTI and ETI immunity, suppress callose response to elf18	Niu et al. 2016
Mg16820	*M. graminicola*	Rice	ascorbate peroxidases (APXs); Reduce ROS, suppress PCD, increase susceptibility	Lin et al. 2016
MeTCTP	*M. enterolobii*	Tobacco	Suppress PCD	Zhuo et al. 2017
MiISE6	*M. incognita*	Arabidopsis	Suppress jasmonate signaling	Shi et al. 2018
Mi-cm-3	*M. incognita*	Tobacco	Suppress salicylic acid (SA) mediated immunity	Wang et al. 2018
Mg01965	*M. javanica*	Rice	Suppress immunity, suppress plant defense	Zhuo et al. 2019
MjMIF	*M. javanica*	Tobacco, Arabidopsis	Annexins; Reduce H₂O₂ levels, suppress immunity	Zhao et al. 2019
MiEFF18	*M. incognita*	Arabidopsis	SmD1; Giant cell formation,	Mejias et al. 2021
MiPDI1	*M. incognita*	Tobacco, Arabidopsis	Stress Associated Protein (SAP); Suppress immunity, giant cell formation	Zhao et al. 2020
MiCTL1	*M. incognita*	Tobacco, Arabidopsis	Catalases (CAT2/3); Reduce H₂O₂ levels, suppress immunity	Zhao et al. 2021
Mg-PEL1	*M. graminicola*	Rice	Cell wall; activate plant defense, PCD, ROS accumulation	Chen et al. 2021
Minc03329	*M. incognita*	Tomato, Arabidopsis	Suppress flg22-induced PTI, Suppress immunity	Zhou et al. 2023a, b)
Mi2G02	*M. incognita*	Arabidopsis	trihelix transcription factor GT-3a, protolysis; Giant cell formation	Zhao et al. 2024
MiEFF12	*M. incognita*	Tobacco,	Solanum lycopersicum plant bap-like (PBL), and basic leucine zipper 60 (BZIP60)	Soulé et al. 2024
MiCE108	*M. incognita*	Tobacco, Arabidopsis	cysteine protease RD21A; Suppress immunity	Yu et al. 2024
MgCRT1	*M. graminicola*	Rice	pathogenesis-related protein OsPR1#101	Yang et al. 2025
MiV86	*M. incognita*	Tobacco	RING finger protein 217, RBR-type E3 ubiquitin ligase, NbRNF217; Suppress NRC-4 related immunity	Qin et al. 2025
MiMSP8	*M. incognita*	Tomato	U2 snRNP auxiliary factor (U2AF), disrupt pre-mRNA splicing	Chen et al. 2025

## Major root-knot nematode management strategies

Managing root-knot nematodes remains a challenging task in modern agricultural practice. Successful management requires integration of multiple strategies to keep nematode populations below agronomic damage thresholds (Abd-Elgawad 2025). The following describes the current major root-knot nematode management strategies.

### Chemical nematicides

Due to environmental and health concerns, the use of chemical nematicides has become increasingly restricted, but remains a relatively fast and effective tool for large-scale agriculture. Chemical nematicides are broadly classified into two groups: fumigants and non-fumigants. Fumigants are traditionally volatile compounds that diffuse into soil pores and kill nematodes via the toxic vapor. Common examples include 1,3-Dichloropropene (1,3-D, Telone^®^), Chloropicrin, Methyl Bromide, Metam salts (Vapam^®^, K-Pam^®^) and Dazomet (Basamid^®^). Fumigants act quickly and provide broad-spectrum control of nematodes, insects, weeds, and fungi. However, their high non-target toxicity often kills beneficial soil flora and fauna, disrupting the healthy soil ecosystem and posing risks for nematode resurgence. In addition, fumigants are known to be toxic to vertebrates; therefore personal protective equipment (PPE), specialized training with standard operating Procedure (SOP) are required for human applicators. Fumigants may also have the potential to contaminate groundwater and air if not applied according to label specifications, thus they are regulated as VOC-emitters and ozone-depleters in some regions. US EPA regulates the federal baseline of fumigant application, and many states have their own restrictive regulations or have banned their use (USEPA 2025).

Chemical nematicides classified as non-fumigants are applied as seed treatments or are applied in-furrow at planting. Non-fumigants such as Fluopyram (Copeo^®^, Velum Prime^®^, Velum One^®^), a type of succinate dehydrogenase inhibitor (SDHI), is both a nematicide and a fungicide that can be used as a seed treatment or in-furrow, to disrupt mitochondrial electron transport and energy metabolism of nematodes. Another non-fumigant Fluensulfone (Nimitz^®^), applied to soil in a pre-planting window, is nematicide-specific; it disrupts nematodes’ feeding, egg-laying, and locomotion, causing starvation and death. Fluensulfone is not neurotoxic, but its action mechanism has not been fully understood. The organophosphate nematicides often applied in-furrow such as Fosthiazate (Nemathorin^®^), Oxamyl (Vydate^®^) and Aldicarb (AgLogic^®^) inhibit acetylcholinesterase at the synapse of neurotransmission, are neurotoxic, thus face increasing regulatory restrictions and are being phased out in many countries (Noling 2016). Compared with fumigants, non-fumigants like fluensulfone and fluopyram are generally considered to have lower environmental and health impacts and are less destructive to the soil microbial community (Morris et al. 2016; Desaeger et al. 2020). However, non-fumigant nematicides are generally more expensive, and their applications are unfeasible for many growers unless economically viable, and repeated use can lead to the development of resistance. Due to their adverse environmental and health impacts, organophosphate and carbamate nematicides have become increasingly restricted, which has led to a further reduction in the number of available nematicides (Chen et al. 2020). In addition, the narrow pre-plant application window for nematode management often fails to coincide with peak nematode activity later in the growing season, which can limit the effectiveness of chemical control measures. Furthermore, non-target effects may disrupt beneficial soil microbiota, particularly mycorrhizal fungi essential for plant nutrient uptake (Entry et al. 2002).

### Biological control

Biological control using microorganisms (bacteria, fungi) to suppress nematode population and provides a sustainable alternative to the broad-spectrum chemical nematicides. They act through various modes, including parasitism, toxin production, competition, and induction of plant host resistance. For example, *Bacillus spp*. is one of the common commercial bacterial biocontrol agents found in products like Votivo^®^, BioST^®^, and Aveo^®^ EZ. They act through the release of nematicidal toxins (e.g., surfactins, iturins) and enzymes (e.g., chitinases, proteases) to lyse nematode eggs and cuticles. Nematode egg-parasitic fungi (*Purpureocillium*, *Pochonia*) are also formulated in products such as MeloCon^®^ WG, BioAct^®^, and Rizotec^®^. These fungi produce a specialized parasitized structure named appressoria that can penetrate the nematode eggshell and affect female reproduction (Manzanilla-López et al. 2013). However, environmental sensitivity to UV radiation and temperature fluctuations necessitates frequent reapplications of these products. Commercial biocontrol agents like *Purpureocillium lilacinum* and *Bacillus firmus* displayed efficacy in RKN control but also showed inconsistent results due to soil compatibility issues (Isaac et al. 2021, Ghahremani et al. 2020; Gattoni et al. 2023; Khan and Tanaka 2023).

Regulatory barriers have stalled next-generation RNAi products as biopesticides, primarily due to concerns about dsRNA persistence in soil. Comprehensive assessments of their environmental safety and the knowledge regarding potential exposure risks to non-target organisms are still lacking (Zarrabian and Sherif 2024). Efficacy of biological control varies widely due to their dependence on soil conditions (temperature, moisture, pH, organic matter, native microbial community, etc.), as they must be applied into the root layer of soil where they can encounter nematodes. While biological controls may not provide the level of efficacy as traditional chemical nematicides, they are an emerging alternative and have shown effective suppression under some controlled conditions. Further studies are needed to assess their efficacy in production agriculture and as a tool for sustainable agriculture, which reduces environmental and health impacts.

### Crop rotation

Crop rotation is a conventional practice to manage RKNs. By rotating susceptible host plants with non-/poor hosts or resistant varieties, the RKNs are starved, and the reproduction cycle is disrupted, thus controlling population density below damaging levels (Trivedi and Barker 1986; Zhang et al. 2025a, b). Since different RKN species have different host ranges, the knowledge of local RKN species, their initial population density, and their corresponding host and non-host species is critical for the selection of correct rotation crops. For example, to control southern root-knot nematodes *M. incognita*, non-/poor host crops including Corn (*Zea mays*), Wheat (*Triticum aestivum*) Sorghum (*Sorghum bicolar*), Oats (*Avena sativa*), Barley (*Hordeum vulgare*), and Pearl Millet (*Pennisetum glaucum*) are suggested (McSorley 2001). Biofumigant plants are not only non-hosts but also release nematicidal chemicals that inhibit juvenile viability and egg hatching, including *Marigold (Tagetes spp*.), *Brassicas***(***Brassica spp.*), Mustards (*B. juncea*), radish (*Raphanus sativus*), and rapeseed (Cerruti et al. 2010, Dutta et al. 2019). Brassicas need to be chopped and added to the soil, allowing their glucosinolates to transform into isothiocyanates, a potent group of fumigants. Legumes such as peanut **(***Arachis hypogaea*) are poor hosts for *M. incognita* that infects cotton. They can also fix nitrogen, increasing nitrogen available for the following season’s crop, an additional benefit to their incorporation into a crop rotation sequence (NC State Extension Publications 2023). However, the non-/poor host period of the rotation usually lasts for at least one full growing season and around 2–4 years for high nematode pressure, potentially leading to decreased revenue for growers (Trivedi and Barker 1986). In addition, weed management is critical during non-/poor host culture period. Common weeds that are hosts of RKNs include pigweed (*Amaranthus* spp.), purslane (*Portulaca oleracea*), and nightshade (*Solanum* spp.) (Weed survey 2005). Combining rotation with other strategies, such as solarization or organic matter amendments, can generate greater effects. Emphasis also should be placed on integrated pest management (IPM) strategies, especially when multiple nematode species are present. Effective IPM of nematodes includes the above-discussed strategies (knowledge of soil conditions and nematodes in the field, chemical and/or biological control, crop rotation or other cultural practices) along with integration of resistant (or partially resistant) varieties, to be discussed in the following section.

### Resistant varieties and engineering

The use of resistant varieties is an environmentally sustainable, cost-effective, and highly efficient management method. Traditional breeding programs often source resistance from wild crop relatives that carry native resistant genes (R genes), which are then introgressed into commercial cultivars. For example, in cotton, resistance is polygenic and quantitatively inherited (Cohen et al. 2024). The primary source is the wild species *Gossypium barbadense* (Pima). Breeding programs have successfully introgressed this resistance into commercial Upland cotton (*G. hirsutum*) varieties, which are now widely planted in nematode-infested regions (Yadav et al. 2025). Grafting rootstock is also considered a practical RKN management strategy, especially in vegetable and fruit productions; the root system of a RKN resistant variety (rootstock) that carries Mi gene, can be grafted to the scion of a desirable/high-yielding but nematode susceptible variety, to achieve both RKN resistance and high-yield benefits (Noling 2002; Liu et al. 2015). The Mi-1.2 gene from tomato is the most well-studied and effective RKN R-gene. It is a single, dominant gene that triggers a hypersensitive response, resulting in rapid cell death upon nematodes developing feeding sites (Dropkin 1969a, Vos et al. 1998). Because of its potency, it has been a prime candidate for genetic engineering in other crops. However, Mi genes-mediated resistance is temperature sensitive, turning ineffective at consistently higher soil temperatures of > 28 degrees Celsius (Dropkin 1969b). Although heat-stable Mi polymorphisms are identified, they have not been successfully transferred to commercial cultivars due to the cross-incompatibility (El-Sappah et al. 2019). The *N* genes and *Me* genes in pepper (*Capsicum annuum L.*) are dominant R-genes used in breeding programs against various RKN species (Djian-Caporalino et al. 1999; Thies and Fery 2000). Cystatins are proteinase inhibitors acting as nematode anti-feedants that disrupt the nematode’s digestive process and inhibit female *M. incognita* reproduction. Genes for plant-derived cystatins (e.g., OC-I Delta D86 from rice) are engineered into crops (Urwin et al. 1997; Papolu et al. 2016). In addition, certain *Bacillus thuringiensis* (Bt) toxins (Cry5B, Cry6A) are effective against RKNs. Engineering crops to express these toxins is an effective strategy (Li et al. 2007; Wang et al. 2024).

Traditional breeding programs for nematode resistance are not precise, time- and labor-consuming, requiring years of crossing and selection cycles, the R gene from wild relatives often provides only narrow-spectrum resistance against specific nematode species. Traditional genetic engineering techniques are widely used, as mentioned above, to introduce R genes (e.g., Mi, Bt) and express functional proteins for nematode resistance. However, overly relying on one single resistant gene can result in the buildup of nematode resistance (Mccarville et al. 2017). Nematodes may rapidly evolve to escape R protein recognition. The future of resistant gene engineering likely lies in pyramiding/stacking multiple R genes or combining R-genes with other strategies like the Host-Induced Gene Silencing (HIGS). HIGS is another plant engineering technique using RNA interference (RNAi) (Fire et al. 1998; Koch and Kogel 2014). Plants are engineered with a foreign gene to produce double-stranded RNA (dsRNA), which is then processed into siRNA. When nematodes feed on plants, they receive these siRNAs and activate nematodes’ own RNAi machinery to silence essential nematode genes, causing lethal or sub-lethal effects (e.g., reduce parasitism success). Compared to traditional transgenic engineering, HIGS is advantageous; it’s RNA-based and sequence-specific, and can be well-designed to target genes unique to the nematode of interest. In addition, no foreign proteins are generated in HIGS process. Chaudhary et al. developed transgenic eggplant (*Solanum melongena*) lines that express dsRNA targeting the *Mi-msp-1* gene for *M. incognita* resistance. They achieved downregulation of up to 5.5 fold of the gene transcript in *M. incognita*, indicating the successful delivery of the silencing components to nematodes via ingestion. There is a significant reduction in susceptibility to *M. incognita* by 30–70% as indicated by various phenotypic parameters, including gall and egg mass numbers, and the nematode multiplication factor (MF) (Chaudhary et al. 2019). Recently, Aparecida et al. used the RNAi approach to knock down three *M. incognita* effector genes Minc01696, Minc00344, and Minc00801, and achieved reduced susceptibility to *M. incognita* (Aparecida et al. 2022).

## CRISPR/Cas9 technologies as a solution

Traditional genetic engineering and HIGS still take a long time to develop and face many limitations and challenges; the transgene is randomly inserted into a host genome, resulting in variations in expression levels and the potential to disrupt important host genes. Sometimes, the genomic region carrying the transgene may be linked to undesirable traits (e.g., lower yield and poor quality), which must be carefully selected against. HIGS often provides only partial resistance without constitutive inheritability. Targeting essential nematode genes or parasitism-related nematode effector genes poses strong selection pressure, accelerating the evolution of resistance-breaking nematode phenotypes. For many economic crops, commercially acceptable nematode-resistant varieties are not yet available. As with all GMO plants, the commercialization of transgenic crops with nematode resistance faces significant regulatory hurdles and public acceptance challenges.

The advent of the clustered regularly interspaced short palindromic repeats (CRISPR)/CRISPR-associated protein 9 (Cas9) genome editing technology represents a paradigm shift in the field of genetic engineering, offering an unprecedented level of precision, efficiency, and accessibility (Zhang et al. 2021). Initially developed from the adaptive immunity system of the bacteria *Streptococcus pyogenes* to combat invasive genetic entities, including plasmids and phages (Horvath and Barrangou 2010), the CRISPR/Cas system allows researchers to make targeted modifications to the genomes of virtually any organism. It has become a promising approach for crop improvement to generate resistance to RKNs (Rasheed et al. 2021).

Knocking out host compatibility factors including susceptibility genes (S genes) is generally considered a lower selection pressure on pathogens, thereby reducing the risk of pathogen adaptation (Zaidi et al. 2018; Dutta 2024). This approach aims to disrupt essential host plant factors that nematodes require for infection, essentially turning the plant into a non-/poor host, making it more difficult for nematodes to overcome and develop resistance. Huang et al. were the first to apply the CRISPR/Cas 9 technique to induce targeted mutagenesis to the rice susceptibility gene *OsHPP04* and successfully obtain homozygous lines resistant to *M. graminicola* without compromising growth and other agronomic traits (Huang et al. 2023). Dutta et al. also employed the CRISPR/Cas 9 system to selectively knock out the amino acid permease (AAP6) gene in *Arabidodpsis* and gain homozygous *M. incognita* resistant line with no growth penalty (Dutta et al. 2024). It is expected that in the following decade we will see an upsurge in the development of nematode-resistant varieties using this revolutionary genome editing technology.

### Standard CRISPR/Cas 9 system

**Fig. 2:**
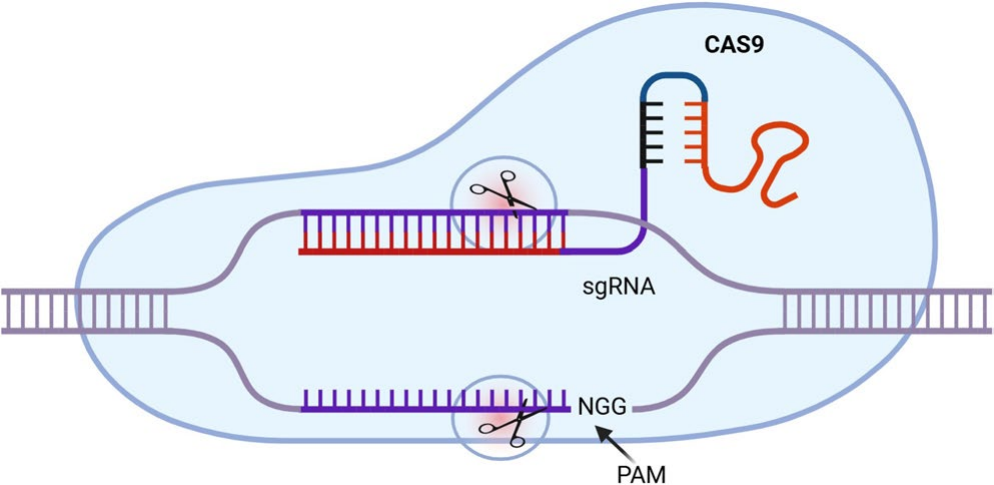
Key elements of CRISPR/Cas 9 system. (Made from Biorender)

The system consists of two main parts: the guide RNA (gRNA) and the Cas 9 nuclease. The gRNA has two components: one is the CRISPR RNA (crRNA), usually 18–20 bp, that recognizes and is complementary to the targeted DNA sequence at a specific genomic locus. Another part is the trans-activating CRISPR RNA (tracrRNA) that is structured as a binding scaffold for holding the whole gRNA-Cas complex. The Cas 9 nuclease contains a recognition (REC) lobe and a nuclease (NUC) lobe, with REC binds to the sgRNA and the NUC (HNH and RuvC) to cleave. The gRNA guides the Cas 9 nuclease to target sites and cleaves the complementary DNA strand about three base pairs upstream of the Protospacer Adjacent Motif (PAM) sequence (Fig. 2).

**Fig. 3:**
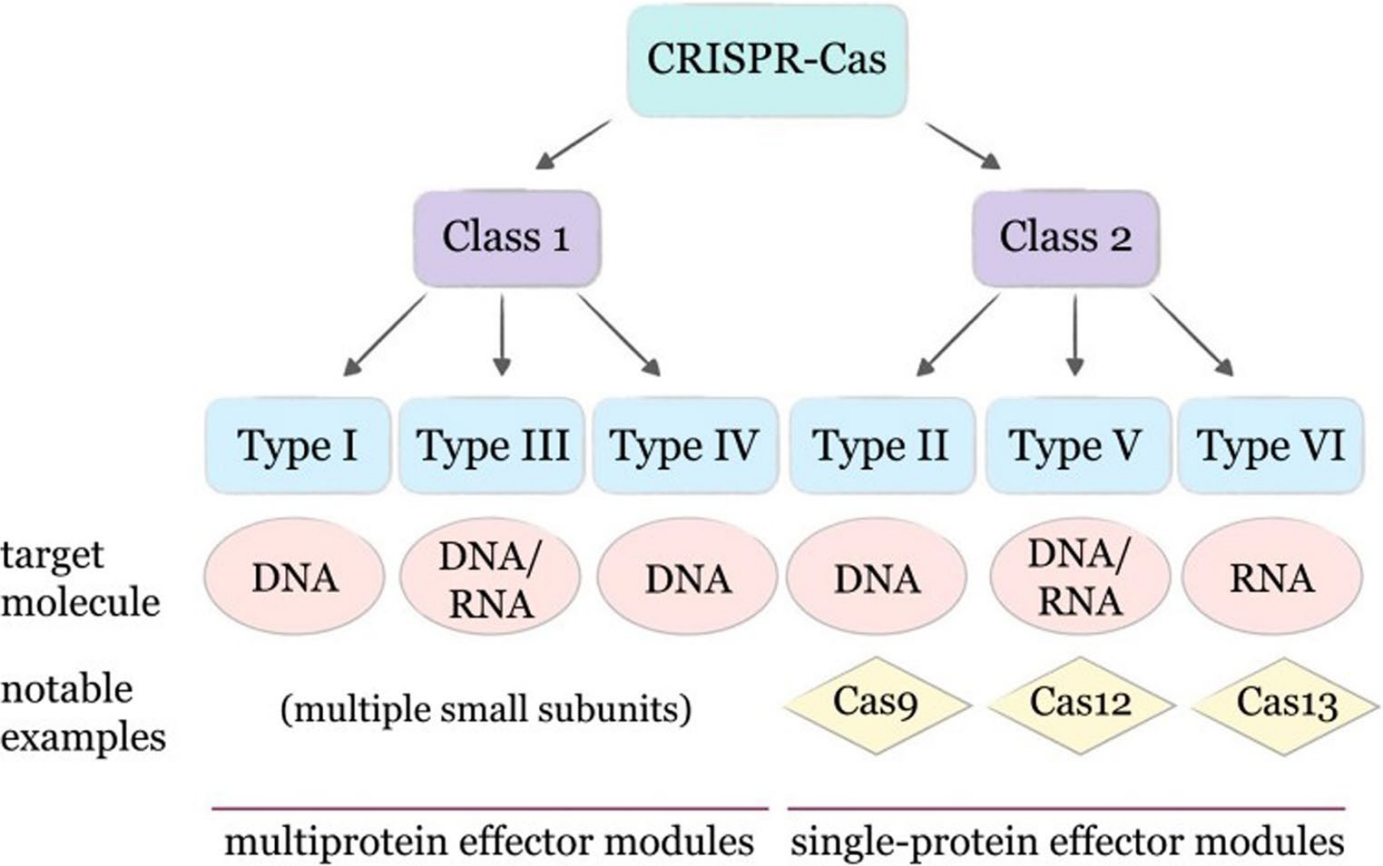
CRISPR/Cas systems classification based on Cas proteins

Based on the sequence similarity, phylogenetic analysis, and functions of Cas proteins, CRISPR/Cas systems are divided into two classes: Class 1 and Class 2. Class 1 systems include types I, III, and IV that use multiple proteins to form a functional complex. In contrast, the Class 2 systems use only one large Cas protein to bind and cleave targeted sequences (Fig. 3) (Makarova et al. 2015, 2020). This makes Class 2 systems easier to construct and deliver into cells from the bioengineering perspective. Different variants of the Cas protein have contributed to a more versatile system, such as the CRISPR/Cas12f with shorter amino acid sequences, providing faster delivery and high efficiency in editing (Liao et al. 2025; Zhang et al. 2025a, b). Among the Cas proteins, Cas9 remains extensively studied and widely utilized in crop genome engineering (Hillary and Ceasar 2023). For example, in cotton genome editing using optimized vectors, ideal targeted mutation frequencies were achieved with no off-target effects detected (Li et al. 2017).

Following gRNA recognition of the targeted site and Cas9 cleavage, cells use two distinct mechanisms for DNA damage repair: the nonhomologous end joining (NHEJ) and the homology-directed repair (HDR). The NHEJ mechanism rapidly rejoins the broken ends, which is fast and efficient but error-prone, usually resulting in random small sequence insertions or deletions (indels). This usually causes frameshift mutations and premature termination of translation, resulting in a gene knockout, which is a desirable outcome in many cases. One disadvantage is that NHEJ pathway generates heterogeneity due to random indels. For more precise edits, such as introducing a specific point mutation or inserting a new DNA sequence, the HDR pathway is utilized. This requires the co-delivery of a DNA donor template containing the desired edit flanked by homologous arms. In short, NHEJ is more efficient for targeted gene knockout, while HDR achieves more precise editing.

### Base editing and prime editing

Base editing and prime editing are more recently developed CRISPR techniques. Base editing directly converts one nucleotide base into another at the targeted genomic locus without causing a DNA double-strand break (DSB) (Komor et al. 2016), while prime editing not only avoids the DSB, it also directly “writes” the new genetic information into the targeted site without using a donor DNA template. Both optimized editing techniques use a modified Cas nickase (nCas9) or deactivated Cas9 (dCas9) that cuts only one strand of DNA or does not cut the targeted DNA to avoid DSB (Anzalone et al. 2019). This reduces the formation of undesirable indels, thus largely offsets the major drawback of the standard CRISPR/Cas9 editing.

Base editing uses a standard guide RNA and the modified dCas9 (may also nCas9) linked to the enzyme deaminase to change a specific DNA base to another (C to T, G to C, A to G, T to C). The Cytosine Base Editors (CBEs) convert C-G pair to T-A, while the Adenine Base Editors (ABEs) convert an A-T pair to a G-C (Gaudelli et al. 2017). In contrast, prime editing uses a special Prime Editing Guide RNA (pegRNA), which is an extended RNA that contains the template for inserting desirable sequences. The prime editor protein is a fusion of Cas 9 nickase and a reverse transcriptase to synthesize the designed DNA sequence (“write”) using the pegRNA carried template to the 3’ end of the nicked DNA. Base editing is relatively simple and more efficient, an ideal tool for correcting point mutations, while prime editing is more versatile in that it can make all 12 possible base-to-base changes, as well as more precise by introducing desirable small indels to targeted genome loci without creating DSB (Anzalone et al. 2020).

## Procedure of CRISPR/Cas9 genome editing for nematode resistance in cotton

An example pipeline for cotton (*G. hirsutum*) genome editing for nematode resistance by knocking out a putative plant compatibility gene is described using a modified procedure based on a previous report (Li and Zhang 2019).

### Step 1- Targeted gene selection, sgRNA design, and vector construction

First, carefully select a plant compatibility gene and identify the gene ID from CottonGen (https://www.cottongen.org). Screening gRNAs within the entire cotton genome using the CHOPCHOP software. The website ranks the gRNA candidates according to their on-target efficiency, mismatch, and off-target potentials. The target must be adjacent to the PAM sequence (5’-NGG-3’), essential for SpCas9 recognition and cleavage. The PAM requirement is the first filter. gRNAs with a guanine (G) at the position immediately preceding the PAM and a moderate GC content (e.g., 40–60%) are generally associated with higher cleavage efficiency. Usually, a gRNA is preferred to be designed at the 1 st or 2nd exon region of a targeted gene; two gRNAs are designed for enhancing genome editing efficiency and later CRISPRized plant selection.

### Step 2 -Assembly of CRISPR constructs via Golden Gate Cloning

There are many ways to construct gRNA into DNA constructs, here is an example. This procedure uses two vector systems to assemble and deliver multiplex CRISPR gRNA constructs into cotton. The initial phase of construct assembly employs the pCBC vector system as an intermediary cloning platform. This system is specifically engineered for the rapid and efficient assembly of multiple DNA fragments using Type IIS restriction enzymes, known as the Golden Gate cloning. This mechanism generates unique, non-palindromic overhangs, allowing directional and seamless assembly of several DNA fragments in a single, one-tube reaction with high accuracy and efficiency. The pCBC vectors serve as entry clones, each harboring individual gRNA sequences flanked by the appropriate BsaI recognition sites, preparing them for the final assembly reaction. Following the Golden Gate assembly reaction, the product is transformed into the competent *Escherichia coli* strain DH5α for amplification, selection, and subsequent plasmid isolation.

### Step 3 - Final assembly and initial verification in a plant binary vector

The final multiplex gRNA construct is transferred into the plant binary vector pKSE401 containing a kanamycin resistance cassette. A second Golden Gate reaction is performed to transfer the gRNA array from the pCBC intermediate into the pKSE401 backbone. The reaction mixture contains high fidelity buffer, BsaI enzyme, the purified pCBC assembly products, and the linearized pKSE401 destination vector. A no-template control (NTC) is included for each reaction to monitor contamination. A thermocycling program will be used to drives the BsaI enzyme to repeatedly cut the DNA fragments, liberating the gRNA inserts with specific overhangs that directionally ligate into the complementary overhangs of the digested pKSE401 vector.

### Step 4 - Primary selection in *E. coli* and molecular verification of constructs

Upon completion of the Golden Gate reaction, the mixture contains a heterogeneous pool of molecules: the desired pKSE401-gRNA construct, non-ligated or re-ligated empty pKSE401 vector, and various intermediate products. This mixture is then introduced into competent DH5α *E. coli* cells via heat shock transformation. Transformed cells are then plated onto LB agar solid media supplemented with kanamycin. Only bacteria that have successfully taken up a plasmid containing the kanamycin resistance gene can survive and form colonies.

After incubation, first-appearing colonies are selected for inoculation in liquid LB media with kanamycin selection. The primary screening method is PCR followed by gel electrophoresis. The objective is to identify clones containing an insert of the expected size within the pKSE401 backbone. The PCR products are then purified and subjected to Sanger sequencing for confirmation.

### Step 5 – Agrobacterium-mediated transformation

Once the sequencing verifies pKSE401-gRNA plasmid, the next step is to introduce it into the plant transformation competent bacterium, *Agrobacterium tumefaciens*, e.g. strain EHA105. This disarmed strain retains its natural ability to transfer T-DNA into plant cells. The transformation is again performed using a heat shock protocol with competent EHA105 cells. Successful transformants are selected on solid media plates containing antibiotics, generating a stable *Agrobacterium* strain harboring the final CRISPR construct. Then, positive *A. tumefaciens* clones are selected for stable transformation, subculture transformed explants, and to perform antibiotic selection.

### Step 6- Evaluation of CRISPR/Cas9 induced mutagenesis

There are many methods for identifying CRISPRized events. Common methods include the T7 endonuclease assay, PCR, and sequencing.

## Limitations of CRISPR/Cas technology

While CRISPR/Cas technology holds immense promises for developing plant nematode resistance, it faces challenges and limitations that must be addressed, including off-target effects, transformation and delivery difficulties, and regulatory issues (Zhang et al. 2019; Li et al. 2025). Many plants, such as upland cotton is tetraploid, have a large and repetitive genome; guide RNAs designed to target susceptible nematode genes may unintentionally edit other genes of the same family, leading to undesirable phenotypes. Improved high-fidelity Cas variants and carefully designed guide RNAs can mitigate off-target effect, but not eliminate it entirely, thus the knockout lines must be thoroughly screened for off-targets (Guo et al. 2023; Li et al. 2025).

Another major technical bottleneck is the delivery of CRISPR components into the plant cell and then regenerating a whole plant. Some important crops are recalcitrant to *Agrobacterium*-mediated transformation, the standard method for CRISPR components delivery. After delivery, the integration and expressions of CRISPR components, and regeneration of a whole plant from transformed cells in explants remains a challenging task warrants future optimizations (Altpeter et al. 2016). The delivery and regeneration through conventional plant tissue culture and the development of advanced technologies, in this field is critical to unlock CRISPR potential in developing nematode resistant crops (Maher et al. 2020). Development of tissue culture-free transgenic and genome editing systems, such *de novo* induction of meristems, virus-induced gene delivery and grafting-based genome editing (Li et al. 2026) will enhance the application of CRISPR genome editing on creating NKZ-resistant new germplasm.

## Law and regulation

There is a global trend to exempt Site-Directed Nuclease type 1 (SDN-1) products from strict GMO regulations and to minimally regulate SDN-2. The rationale lies in the fact that the precise site mutations from the products of SDN-1/2 are indistinguishable from those obtained from natural mutation or conventional breeding. SDN-1 process relies on the cell’s own NHEJ repair pathway to create small mutations and gene knockouts, and the final products do not carry foreign DNA. The SDN-2 process uses a DNA template to guide the repair of the break via the HDR mechanism, resulting in small, specific changes. The final products do not carry a functional foreign gene (Podevin et al. 2013). The USDA’s revised “SECURE” rule exempts most SDN-1 and some SDN-2 plants (USDA 2020) from pre-market scrutiny. However, the regulation varies from country to country, and products resulting from SDN-1/2 are scrutinized on a case-by-case basis in many countries. In contrast, SDN-3 process inserts a large, entire gene (transgene) into the genomic break site, like traditional genetic engineering, so it is still subject to GMO regulation.

## Future perspectives

CRISPR/Cas-mediated genome editing is a precise and powerful technology for developing resistant crop varieties against root-knot nematodes. Recently, proof-of-concept works in model systems, and the commercial plant rice have yielded positive outcomes, mainly utilizing the NHEJ DSB repair pathway (SDN-1), which could be exempted from the GMO regulation. In the coming decade, many breakthroughs in this field are expected.

As RKNs hijack plant developmental pathways to create specialized feeding cells, future strategies could focus on disrupting upregulated genes in the feeding sites and master transcriptional regulators essential for giant cell formation. RKNs reprogram plant metabolism and physiology by using an array of effectors. Since many plant compatibility factors and susceptible genes, including those in plant hormone crosstalk hubs, have been identified for corresponding effectors, which would also be promising targets for CRISPR. Recently, Machine learning (ML)-based prediction of susceptible genes from effector-host interactomes is an emerging and promising paradigm for CRISPR target selection in developing nematode-resistant plants (Sperschneider 2020; Pan et al. 2022). Moreover, since many wild relatives of crops possess potent natural resistance to RKNs that are absent in cultivated varieties, instead of conducting the lengthy traditional breeding process, CRISPR can be used to precisely edit the alleles in commercial cultivars to match the resistant alleles in their wild relatives. Especially, the pangenome-guided identification of resistance alleles from wild relatives is a promising strategy for CRISPR-based technology for developing disease-resistant crops (Bayer et al. 2020). In addition, CRISPR can be used to optimize current RNAi-mediated technology; instead of random integration into the genome, CRISPR can precisely guide dsRNA-producing cassettes to specific, secure plant genome loci, providing more consistent expression levels and a safer profile.

The path for the acceptance of genome-edited crop products relies on open and transparent communications initiated by the scientific community to regulatory authorities, farmers, and the public. The knowledge regarding the safety and benefits of genome-edited crops, which are often indistinguishable from their conventionally bred counterparts, as well as their potential risks, should be conveyed not only through scientific publications but also through various outreach activities and channels. Substantial future efforts will need to be put into the transition of laboratory findings to field deployment of these next-generation crops.

## Data Availability

Not applicable.
